# Electromagnetic Induction Aberration: The Possible Mechanism of Tic Douloureux

**DOI:** 10.7759/cureus.255

**Published:** 2015-03-10

**Authors:** Berkley Rish

**Affiliations:** 1 USN, EVMS,Norfolk, VA, Retired Professor Neurosurgery

**Keywords:** trigeminal neuralgia, tic douloureux, etiology, treatment, electromagnetic induction

## Abstract

A theory based on the principles of electromagnetic induction aberration is presented as the possible mechanism of classic trigeminal neuralgia, tic douloureux.

The anatomy of the dorsal root entry zone of the trigeminal nerve at the pons in the proximity of the superior cerebellar artery presents a scenario conducive to the phenomenon of electromagnetic induction. When the action potentials traversing the axons in this zone of the compromised myelin come into juxtaposition with the vascular structure, the criteria for electromagnetic induction are satisfied. The laws of physics governing the phenomenon indicate that a new current, an aberration, would be produced. This could be responsible for the clinical symptoms of tic douloureux.

Other clinical situations with similar features could share this mechanism. This proposed theory, a merger of anatomy, neurophysiology, and the physics of electromagnetic induction, extends the established concept of vascular compression as the etiology of tic douloureux.

## Introduction and background

Since the first described case of trigeminal neuralgia (tic douloureux) in the era of Galen, the condition has been a conundrum [1]. Despite significant progress in the neurosurgical management of this entity, the etiology, mechanism, and pathophysiology remain an enigma. When Dr. Peter Jannetta reported his microvascular decompression (MVD) procedure on the neurovascular collision site, the etiology was assumed to be the mechanical compression of the nerve by the artery [2-5]. Suspecting that other mechanisms may be operative, I began a review of my experience and that of my peers, and a study of the applicable literature.

## Review

The syndrome of classical trigeminal neuralgia, tic douloureux, is distinguished by unique clinical features: sudden, severe, electrical, sprangling pain occurring after a touch stimulus in the trigger zone of the patient’s face. There is a paucity of sensory deficits and a refractory period may follow a sequence of the shocking pains [1, 6-7].

Secondary trigeminal neuralgia is distinguished by features of neuropathy: hypesthesia, dysesthesia, sensory deficits, and the lack of the triggering phenomenon. This disorder is secondary to intrinsic pathology of the nerve (neoplasm or demyelination) or extrinsic compressing lesions (cysts, neoplasms, or bone pathology) [1, 6-7]

The literature on tic douloureux is replete with electrical terminology: ectopic generation, ephaptic firing, hyperexcitability, hyperpolarization, spontaneous synchronization, cross-excitation, electrogenesis, ignition theory, mini-seizure phenomenon, etc., all terms suggesting erratic electrical activity [8-14].

The medical treatments with any modicum of success have been the use of anticonvulsive agents, such as Dilantin or Tegretol. The action of these pharmacological agents alters the electrical properties of the neuron [1, 6-7].

Surgical treatment of tic douloureux has been the destructive interruption of the afferent axons of the trigeminal nerve by various means. These procedures produce relief but leave functional deficits [6, 15-17]. The history of these surgical efforts includes many of the early giants in neurosurgery: Hartley, Krause, Horsley, McEwen, Frazier, Spiller, Cushing and Dandy. The target of the procedures progressed from the peripheral branches of the trigeminal nerve to the divisions of the Gasserian ganglion, to the ganglion itself. As advancements in surgical technique, anesthesia, and the control of hemorrhage permitted, the posterior fossa was entered and surgery was performed on the retrogasserian portion of the trigeminal nerve to its entry into the pons. To preclude the morbidity of surgery, many other destructive modalities were used. A common axiom soon became apparent. If the afferent axons were interrupted or damaged, the tic symptoms would cease. The tradeoff was loss of the axon function. When Dr. Jannetta performed his MVD procedure on the dorsal root entry zone (DREZ) of the trigeminal nerve, the axons’ structure and function were preserved [2-5].

It was then appreciated that both the impulse current (afferent action potentials) and the juxtaposition of a vascular structure were necessary components of the tic syndrome. This scenario had been suggested by Dr. Walter Dandy in 1925 [17].

Questions arose when post-mortem studies on a general population revealed a significant number of the deceased had trigeminal DREZ neural/vascular juxtaposition and no history of tic douloureux [1, 6-7, 18]. Was the myelination of the DREZ sufficient to preclude tic douloureux in those cases?

Dr. Jannetta reported the phenomenon of the symptoms of hemifacial spasm stopping and starting repetitively, as he performed an MVD procedure on the facial nerve. The anesthesiologist recorded the drama, and Dr. Jannetta explained that the events coincided with his separating the artery from the nerve and then simply repositioning the structures without compression or deformity. This was objective evidence that an invisible surreal factor existed, other than mechanical anatomical compression [19].

With these factors established, a review of the unique features of the neuron and the blood vessel was necessary.

To study the electrogenesis of the neuron, the classic paper by the Nobel laureate, Dr. Bernard Katz, was reviewed. His work, re-published in a commemorative issue of Scientific American, delineates the unique electrogenesis capacity of the neuron and the propagation of the impulse along the axon [20]. This concept applied to the trigeminal nerve would account for a current source, the afferent action potentials cascading along the axons of the nerve from one node of Ranvier to the next. The origin of the current would have been the touch stimulus to the trigger zone in the patient’s face. This mechanical stimulus would, by the process of transduction, initiate the selective exchange of ions (Na, K, Cl) across the membrane between the intra- and extracellular spaces, creating a differential in the balance of charges in the two spaces, a current. This current begins a self-propelling cascade along the cellular membrane as the charges seek to neutralize the differential. Arriving in the cell body of the neuron, the charges build to a critical level and initiate an “all or none” firing of a renewed impulse current, which conveys toward the end bulb area of the afferent axon. In the presynaptic end bulb area, enzyme enhanced chemical reactions ensue to transport the charge across the synaptic gap to the awaiting end bulb of the axon of the next neuron in the sequence.

In the case of tic douloureux, this current, an action potential cascade, must traverse the DREZ of the trigeminal nerve at its entrance into the pons. This transition zone between central and peripheral nerve structure is naturally deficient in myelin protection [9-10, 12-13, 18].

To study the role of the blood vessel, a 1955 lecture/demonstration at Wake Forest University School of Medicine by Dr. Merrill Spencer and Dr. Adam Dennison, professors of physiology, was recalled. They had invented a vascular flow meter using the basic physics of electromagnetic induction, as described by Faraday and Maxwell. The aberration observed when an intact blood vessel was placed in the electromagnetic field of their instrument was calibrated to reflect the velocity and volume of the blood flow [21]. The similarities between the anatomical and physiological features of tic douloureux and the principles used in the vascular flowmeter were noted.

Blood conveying in a vessel is a slurry of components, including salt solutions (Na, K, Cl), proteins and cellular elements, leukocytes, and iron laden erythrocytes. These components have varying capacities of conductivity. The composite solution is a random mix slurry pulsating along in a constant flowing motion inside a thin-walled conduit [22].                 

Further review of the physics of electromagnetic induction revealed that the 19th century was a period of discovery in the science of electricity. Oersted linked electricity and magnetism (1820), describing them as two facets of the same phenomenon, like two sides of a coin, each with the capacity to create the other. In 1826, Ohm defined the relationship of voltage, current and resistance (V=IR). Faraday discovered electromagnetic induction in 1831 and published his law, which states that a changing electromagnetic field will induce a current in a nearby conductor if motion is present. He further described how this new current (inductance) would be shared throughout the conductor (self-inductance), into the source of the original current (mutual inductance), and into contiguous conductive structures (transferred inductance) [23-26].

Maxwell provided the mathematical equations for electromagnetic induction in 1873 when he published the four equations (laws) of electromagnetic induction, which bear his name. The first describes the shape and strength of the electrical field generated by a charged object and states that the strength of this field is inversely proportional to the square of the distance from the measuring point to the surface of the object (Gauss’s law). Maxwell’s second equation describes the magnetic field lines around the dipoles of a magnet, stating that these lines are always closed loops running from a north to a south pole of the magnet. The third and fourth equations, reciprocals of each other, state that a changing electric current produces a magnetic field and that a magnetic field produces an electric current. This last equation is a restatement of Faraday’s law of electromagnetic induction -- when a conductor is in motion in the electromotive field of an electric current, their respective force field lines intersect and produce a new current, an aberration. Thus when the four criteria -- current, conductor, proximity, and motion, are satisfied, electromagnetic induction will ensue and be shared according to the established laws of physics [23-26].

The questions are apparent. Does the scenario presented by the trigeminal nerve and the superior cerebellar artery satisfy the four criteria required for electromagnetic induction? Could an electromagnetic induction aberration (EMIA) be the mechanism of the symptoms of tic douloureux?

The electric current criterion is satisfied by the action potentials conveying along the axons of the trigeminal nerve. This current is small, but not tiny. No minimum limit of current required to incite magnetic induction in a conductor has been established. The proximity criterion is satisfied when the superior cerebellar artery is in juxtaposition with the DREZ of the trigeminal nerve. The blood vessel satisfies the conductor and motion criteria. With the criteria satisfied, electromagnetic induction should occur. According to the laws of physics regarding inductance sharing, the new current, an aberration, would convey throughout the blood vessel and back into the current source. It would then follow the established pathways through the mesencephalon to the contralateral thalamic nuclei and finally to cortical foci. The aberration, a result of the EMIA event that occurred at the DREZ, would be different from the original touch signal action potentials and could be a nociceptive sprangling electrical storm. The patient might experience this as the horrid pain complex of tic douloureux. The presumed shift of the touch sensation, carried by the large caliber fast conducting axons, to nociceptive pain sensation, usually carried by the smaller slower conducting axons, has been attributed to cross firing between poorly myelinated hyperexcited axons, without consideration of EMIA [9]. However, the aberration signal storm, now interpreted by the cortical neurons as a nociceptive sensation, could have been conveyed by any axon and a shift would be a moot point or a chance result of the anatomical orientation of axons and the artery contact point at the DREZ.

To examine this theory, the clinical features of tic douloureux and the results of the various treatment modalities were reviewed with particular emphasis being placed on the four necessary criteria for electromagnetic induction.

When the electric current criterion is interrupted or modified by surgical interruption, compression, chemical lysis, thermal lesions, or focused gamma radiation, the tic symptoms are arrested [1, 6-7]. Effective medications, such as anticonvulsants, impact the electrical parameters of the neuron altering the current criterion [1, 6-7]. 

If the proximity criterion is removed, the tic symptoms are arrested, and in Dr. Jannetta’s MVD procedure, this is accomplished without destroying the function of the axons involved. The necessity of the proximity factor was further demonstrated by Dr. Jannetta’s dramatic stop/restart display during surgery on the facial nerve [2-5, 19].

Non-conducting lesions compressing the DREZ, such as epidermoid cysts, benign tumors, and bony lesions, fail to satisfy the conductor and motion criteria. These patients present with neuropathy, not tic douloureux [1, 6].

Interventional neuroradiologists reported the resolution of tic douloureux after the remote endovascular embolization of an arteriovenous malformation (AVM) of the superior cerebellar hemisphere, which was impinging on the trigeminal DREZ. The AVM was left in place, but the embolization had arrested the pulsatile movement of the blood. The motion criterion was removed [27].

A Vietnamese girl was struck by a long-range bullet, which entered her cranium posterior to her right ear. She presented aboard the hospital ship, USS Repose, with neuropathy of her face but no tic pain. At surgery, the bullet was found entangled in the rootlets of the trigeminal nerve at the DREZ and removed without incident. The bullet was a copper-jacketed steel slug, an excellent conductor, but tic symptoms had not occurred; only neuropathy presented. With the bullet at rest, the motion criterion was not satisfied.

Patients with multiple sclerosis are particularly prone to have tic douloureux and post-mortem examinations reveal demyelinating plaques in the DREZ of the trigeminal nerve [19, 28]. Such a feature would abet the proximity criterion by depleting the myelin insulation protection of the axons from any vessel in the area. Electron microscopy of axons from patients with tic douloureux have shown similar demyelination and regeneration at the site of vascular compression [19, 28]. These changes, along with microneuromata and scarring, have been found in Gasserian ganglion tissue from patients with tic douloureux and no multiple sclerosis [18, 28].

Other syndromes may meet these criteria and could share the electromagnetic induction aberration etiological premise. The list would include seizures associated with AVM’s or vascular tumors; post-traumatic epilepsy; other cranial nerve syndromes as well as lateral medullary syndrome (currently being investigated by Dr. Jannetta); peripheral nerve injuries; and classical causalgia as described by Dr. Weir Mitchell based on his Civil War experience. He eloquently described the anatomy of the wounds noting the association of an injured nerve and a large artery. The delay in the onset of causalgic symptoms conforms to the lag time phase of the ensuing demyelination following injury. The surgical treatment consisted of separating the nerve from the vessel. This would have been an early historic example of a macrovascular decompression procedure removing the proximity criterion [29-30].

The passage of an electromagnetic induction current into myelinated axons has been studied by Dr. Bradley Roth, Professor of Physics, Oakland University, Rochester, MI. Further study of this premise in a tic douloureux scenario by physicists and neurophysiologists would validate, or refute, the theory of etiology under consideration [31].

## Conclusions

The theory of electromagnetic induction aberration is radical and heretical. In the past fifty years, significant progress has been made in microsurgery, neurophysiology, enzyme chemistry, intracellular and trans-synaptic chemistry and electron microscopy. During this era, however, the electrogenetic property of the neuron and the conductivity of blood vessels may have been overlooked.  

Electromagnetic induction aberration will occur if a nerve (current source) with inadequate myelin protection is in the proximity of a blood vessel (conductor in motion). This theory, a merger of anatomy, neurophysiology, and the physics of electromagnetic induction, presents a mechanism for the symptoms of tic douloureux and extends the established principle of vascular compression as the etiology of tic douloureux.

Research, particularly by neurophysiologists and physicists, to validate or refute this theory will advance our understanding of tic douloureux and promote better therapeutic design.
